# Genome-Wide Analysis and Expression Profiling of Soybean *RbcS* Family in Response to Plant Hormones and Functional Identification of *GmRbcS8* in Soybean Mosaic Virus

**DOI:** 10.3390/ijms25179231

**Published:** 2024-08-26

**Authors:** Fangxue Zhou, Wenmi Feng, Kexin Mou, Zhe Yu, Yicheng Zeng, Wenping Zhang, Yonggang Zhou, Yaxin Li, Hongtao Gao, Keheng Xu, Chen Feng, Yan Jing, Haiyan Li

**Affiliations:** School of Breeding and Multiplication (Sanya Institute of Breeding and Multiplication), Hainan University, Sanya 572025, China; 21110901000050@hainanu.edu.cn (F.Z.); 22220951310049@hainanu.edu.cn (W.F.); 22210901000010@hainanu.edu.cn (K.M.); 23220951310017@hainanu.edu.cn (Z.Y.); 20223007394@hainanu.com (Y.Z.); 996107@hainanu.edu.cn (W.Z.); ygzhou@hainanu.edu.cn (Y.Z.); yaxinli@hainanu.edu.cn (Y.L.); 184240@hainanu.edu.cn (H.G.); 184093@hainanu.edu.cn (K.X.); 184415@hainanu.edu.cn (C.F.)

**Keywords:** *RbcS* gene family, soybean, genome-wide characterization, expression analysis, soybean mosaic virus

## Abstract

Rubisco small subunit (RbcS), a core component with crucial effects on the structure and kinetic properties of the Rubisco enzyme, plays an important role in response to plant growth, development, and various stresses. Although *Rbcs* genes have been characterized in many plants, their muti-functions in soybeans remain elusive. In this study, a total of 11 *GmRbcS* genes were identified and subsequently divided into three subgroups based on a phylogenetic relationship. The evolutionary analysis revealed that whole-genome duplication has a profound effect on *GmRbcSs*. The cis-acting elements responsive to plant hormones, development, and stress-related were widely found in the promoter region. Expression patterns based on the RT-qPCR assay exhibited that *GmRbcS* genes are expressed in multiple tissues, and notably *Glyma.19G046600* (*GmRbcS8*) exhibited the highest expression level compared to other members, especially in leaves. Moreover, differential expressions of *GmRbcS* genes were found to be significantly regulated by exogenous plant hormones, demonstrating their potential functions in diverse biology processes. Finally, the function of *GmRbcS8* in enhancing soybean resistance to soybean mosaic virus (SMV) was further determined through the virus-induced gene silencing (VIGS) assay. All these findings establish a strong basis for further elucidating the biological functions of *RbcS* genes in soybeans.

## 1. Introduction

Soybean (*Glycine max* (L.) Merr.) is a very important legume crop. It is not only one of the most economically significant oil crops but also serves as a major source of protein and oil in human food and animal feed [1,2]. With the release of the whole-genome sequencing results for cultivated soybeans, we have gained a deeper understanding of this crucial crop, which depends on the exploration and research into gene functions [1,3,4,5]. Currently, genome-wide expression analysis has become a pivotal approach to mining and studying gene functions via identifying and elucidating the biological processes involved in or regulated by specific target genes [6,7,8].

Plants are commonly subjected to a range of response signals during their development [9,10,11]. It is a complex and intricate cascade reaction wherein the perception, transduction, and signal-driven processing of light signals are particularly crucial, ultimately resulting in specific cellular responses [12,13]. This also reflects the key role of photosynthesis in plant growth and development. Chloroplasts are the organelles most intimately involved when plants engage in photosynthesis, and their development is also linked to various fundamental biological processes, such as CO_2_ assimilation [14,15]. Ribulose 1, 5-bisphosphate carboxylase/oxygenase (Rubisco), one of the most abundant proteins and enzymes in the biosphere, is a key enzyme in the Calvin cycle and CO_2_ assimilation process, particularly in C3 plants such as rice, soybean, and various other crops [16,17,18].

Rubisco is a 16-subunit enzyme composed of eight large subunits (RbcL) containing catalytic sites and eight small subunits (RbcS) with certain effects on the kinetic properties [19,20]. Moon and Thompson estimated the molecular weight of the large subunit to be 55,000 Da and the small subunit to be 16,000 Da [21]. The large subunit is encoded by a single gene (*RbcL*) in the chloroplast genome, whereas the small subunit is encoded by a nuclear family of several genes, with members sharing a high amino acid identity [22]. In recent years, researchers have shown increasing enthusiasm for studying RbcS and have proposed that RbcS plays a role in influencing Rubisco’s catalytic efficiency and performance, CO₂ specificity, activity, quantity, assembly, and stability [23,24,25,26,27,28]. These studies suggest that the *RbcS* gene itself may play an important role in maintaining the structural stability and activity of Rubisco.

The *RbcS* multigene family comprises 2 to 22 members, varying depending on the species [24,29]. The functions of *RbcS* in plant growth and development have also been explored. Four genes of the rice *RbcS* multigene family, *OsRbcS2* to *OsRbcS5*, exhibited high levels of expression in the leaf blades, whereas the accumulation of *RbcS* mRNAs in leaf sheaths, roots, and developing spikelets was comparatively low, showing that *RbcS* gene expression is tightly coordinated throughout the lifespan of rice [30]. *RbcS1A* and *RbcS3B* mainly contribute to the accumulation of Rubisco in Arabidopsis leaves, and these genes worked synergistically to produce sufficient Rubisco for the leaf photosynthetic capacity [31]. In the model tetraploid crop tobacco, the introduction of indel mutations across multiple *RbcS* homolog genes led to a remarkable reduction in Rubisco content by approximately 93%. Consequently, the mutant plants accumulated only 10% of the total biomass compared to wild-type plants [32]. Compared with other species, few studies on *RbcS* in soybeans have been reported. The genes encoding RbcS were analyzed on the growth of soybean seedlings under light- and dark-grown conditions [33]. The results showed that the *RbcS* RNA degraded more rapidly in light than in darkness, demonstrating that soybean *RbcS* genes may be subject to control, wherein light-induced expression is accompanied by a higher rate of RNA breakdown [33]. Additionally, the effects of white light, far-red light, and darkness on in vitro transcription and RNA levels of *RbcS* were investigated in petunia and soybeans. In contrast to petunias, large reductions in *RbcS* RNA were seen in mature soybean plants treated with darkness, along with a slight decrease in the soybean RbcS in vitro transcription rate, suggesting a potential role of RbcS in soybean maturity [34]. It is notable that Rubisco has been widely implicated in the processes of both biotic and abiotic stresses, particularly in terms of the crucial roles played by *RbcL* in virus diseases [35,36,37,38,39,40]. As for *RbcS*, Lin et al. (2011) found that the P3 proteins encoded by different virus isolates, including shallot yellow stripe virus onion isolate (SYSV-O), onion yellow dwarf virus (OYDV), soybean mosaic virus pinellia isolate (SMV-P), and turnip mosaic virus (TuMV), could interact with onion RbcS and RbcL, respectively. This finding suggested that the potyvirus P3 protein disrupted the normal functions of Rubisco by influencing RbcS and RbcL, which in turn led to the development of disease symptoms [41]. Recently, Qin et al. (2024) reported that the reduction of *NbRbCS* levels greatly impaired the intercellular movement of four potyvirids, including areca palm necrotic ring spot virus (ANRSV), turnip mosaic virus (TuMV), pepper veinal mottle virus (PVMV), and telosma mosaic virus (TeMV), indicating RbCS was selected by the virus as a scaffold protein to enable viral movement [42].

To date, no comprehensive genome-wide analysis and characterization of *RbcS* in soybeans has been carried out. In this study, a total of 11 soybean *RbcS* genes were surveyed using bioinformatics methods, including gene characterization, phylogenetic relationships, chromosomal location, collinearity analysis, promoter cis-acting elements, and expression analysis among different tissues and in response to various plant hormones. Additionally, the representative gene, *Glyma.19G046600* (*GmRbcS8*), was selected to determine its role in SMV resistance. These findings will lay the foundation for further research on the potential role of RbcS pathways in response to various biological processes.

## 2. Results

### 2.1. Identification and Physicochemical Characterization of GmRbcS Family Members

The Hidden Markov model was initially used to scan the gene domain of the *GmRbcSs* family. Subsequently, based on homologous sequence alignment and conserved domain analysis, a total of 11 *GmRbcSs* gene family members were identified in this study (Table 1). The amino acid (aa) length of GmRbcSs proteins ranged from 90 aa to 178 aa, and the molecular weights (MWs) of the proteins were between 9.97 kDa and 20.02 kDa. The theoretical isoelectric point (pI) varied from 7.81 to 9.81, indicating that the GmRbcSs proteins were weakly alkaline. The instability index analysis revealed that half of the GmRbcS were potentially unstable proteins (with values above > 40), and the other five proteins were possibly stable proteins (with values from 30.71 to 36.75). The grand average of hydropathicity (GRAVY) values ranged from −0.06 to −0.53, suggesting that they were amphoteric proteins. Additionally, the predictions of the subcellular localization of GmRbcSs indicated that all 11 members were located in chloroplasts.

### 2.2. Conserved Motif, Gene Structure, and Domain Analysis

**Figure 1:**
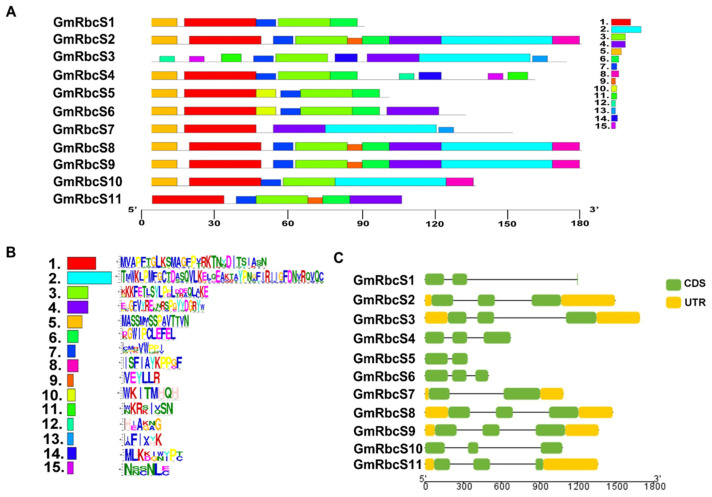
Characteristic of GmRbcS family members. (**A**) Conserved motif analysis of GmRbcSs protein. (**B**) Sequences logos of the identified motifs. (**C**) Exon/intron structures of *GmRbcSs* family members in soybean.

MEME online software (versin 5.5.5) was used to analyze the conserved motif, and the results showed that 15 conserved motifs with amino acid lengths of 2 to 38 aa were identified among the 11 GmRbcSs family members (Figure 1A). Among them, motifs 1, 3, 5, and 7 were prevalent in most GmRbcSs proteins, which demonstrated that GmRbcSs were highly conserved (Figure 1B, Supplementary Table S1). Gene structures of *GmRbcSs* family members were further examined. The distribution of exons and introns was analyzed using GSDS2.0 (https://gsds.gao-lab.org/index.php, accessed on 24 May 2024). All members possessed introns, and the number of exons varied between two and three (Figure 1C). Overall, most *GmRbcSs* members within the close phylogenetic branch exhibited similar structure characteristics in terms of the exon’s number and length. Moreover, to further investigate the structure characteristics, the conserved domain of GmRbcS was analyzed via the NCBI online tool. The results revealed that these proteins primarily exhibit the conserved characteristics of the Rubisco small subunit.

### 2.3. Phylogenetic Construction, Chromosome Distribution, and Collinearity Analysis

**Figure 2:**
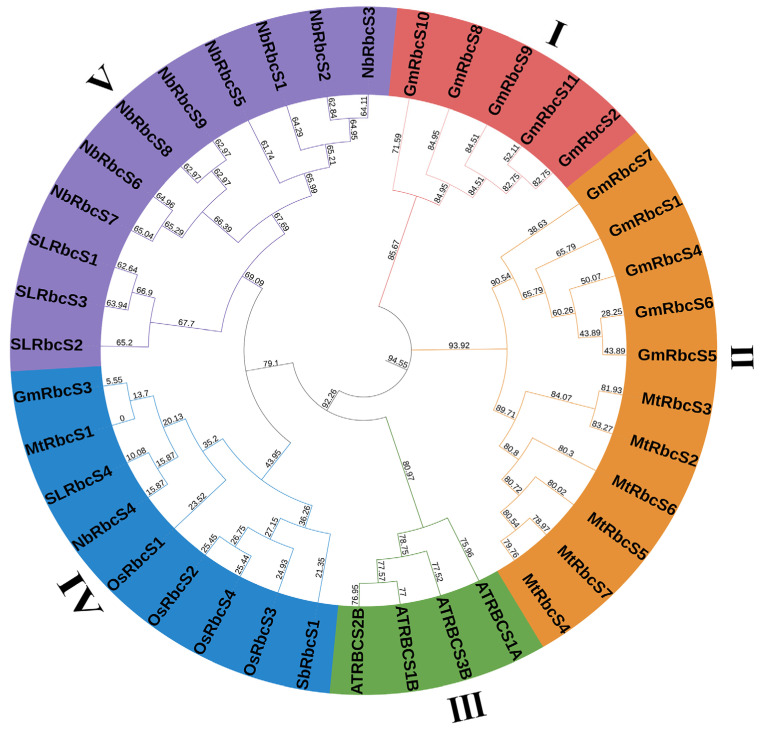
Unrooted phylogenetic tree of RbcSs protein among Glycine max (Gm), Arabidopsis thaliana (At), Nicotiana benthamiana (Nb), Solanum lycopersicum (Sl), Medicago truncatula (Mt), Oryza sativa (Os), and Sorghum bicolor (Sb). The phylogenetic tree was constructed through the neighbor-joining method based on MEGA7.0 with bootstrap values per 1000 replicates. The different colors of the rings represent different subfamilies.

The phylogenetic trees of RbcSs members in soybean, *Arabidopsis thaliana* (*A. Thaliana*), *Nicotiana benthamian* (*N. benthamiana*), *Medicago truncatula* (*M. truncatula*), tomato, rice, and sorghum were constructed to explore the phylogenetic relationship among different species. A total of 40 RbcS proteins found in various species were divided into five clades, and each clade incorporated the homologous RbcSs among different plant species (Figure 2). GmRbcSs were distributed in groups I (five members), II (five members), and IV (one member), which exhibited a closer relationship to *M. truncatula*. This was primarily due to the fact that they were both leguminous and dicotyledonous plants with a higher phylogenetic proximity, indicating that GmRbcSs shared a similar evolutionary history and functional resemblance with the orthologous RbcS proteins present in *M. truncatula*. These results of phylogenetic development analysis could provide a basis for inferring the origin and function of *GmRbcSs*.

**Figure 3:**
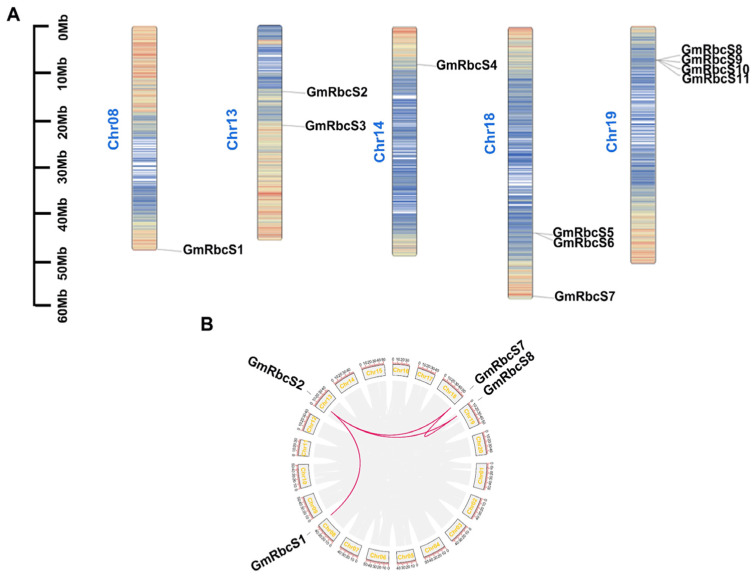
Chromosome distribution and collinearity analysis of *GmRbcSs* family members. (**A**) Chromosomal location of *RbcSs* genes in soybean. The colored rectangular bars represent the chromosomes of soybeans, and the 0–60 Mb scale represents chromosome length. (**B**) Intraspecific collinearity analysis of soybean *RbcSs* family members. The pink lines showed the pairs between genes.

Furthermore, to investigate the genomics distribution and expansion mechanism of *GmRbcSs*, an analysis of chromosome localization and collinearity was carried out, respectively. First, 11 *GmRbcSs* were found to be concentrated on chromosomes (Chr.) 8, 13, 14, 18, and 19 (Figure 3A). Four homologous genes were mapped on Chr. 19, and two homologous genes were mapped on Chr. 18. Tandem duplications may be the reason why these genes are closely located to each other on the same chromosome. Subsequently, a collinearity analysis of GmRbcSs was performed using MCScanX (A plugin in Tbtools, version 1.098). As a result, four genes (*GmRbcS1*, *GmRbcS2*, *GmRbcS7*, and *GmRbcS8*) distributed across different chromosomes showed a significant homology (Figure 3B). Among them, *GmRbcS2* and *GmRbcS8* shared a common pairing with *GmRbcS7*, and *GmRbcS2* was also paired with *GmRbcS1* and *GmRbcS8*. These results indicated that the four genes had a common origin, and they formed four copies based on the twice duplication events of soybean.

### 2.4. Comparative Comprehensive Map of RbcSs in Different Species

**Figure 4:**
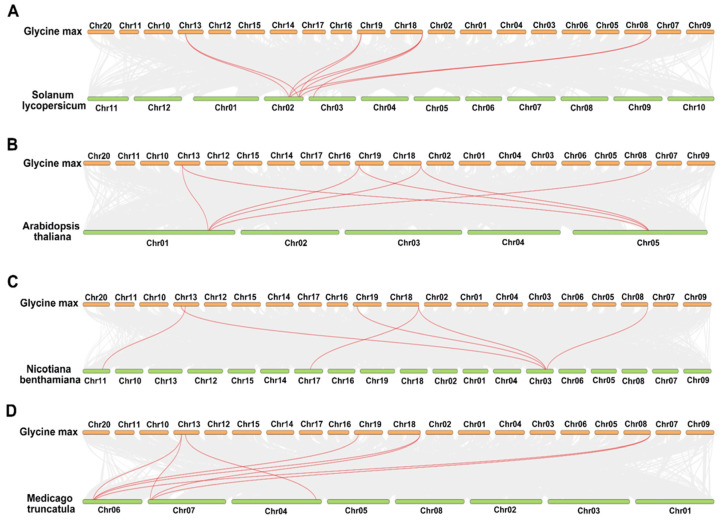
Synteny analysis of *GmRbcSs* family genes between *S. lycopersicum*, *A. thaliana*, *N. benthamiana*, and *M. truncatula*. (**A**) Syntenic analysis of *RbcSs* with the corresponding genes in *G. max* and *S. lycopersicum*. (**B**) Syntenic analysis of *RbcSs* with the corresponding genes in *G. max* and *A. thaliana*. (**C**) Syntenic analysis of *RbcSs* with the corresponding genes in *G. max* and *N. benthamiana*. (**D**) Syntenic analysis of *RbcSs* with the corresponding genes in *G. max* and *M. truncatula*. The red lines showed the gene pairs between two species.

Gene replication plays a crucial role in accelerating the emergence of new gene functions and the expansion and evolution of gene families. Thus, to gain a deeper understanding of how the RbcSs family evolved in soybeans compared to other plant species, the collinearity analysis of soybean, *A. thaliana*, *N. benthamiana*, *M.  truncatula*, and *S. lycopersicum* was constructed, respectively (Figure 4, Supplementary Table S2). The results revealed the existence of homologous relationships between pairs of genes, specifically between four soybean *GmRbcSs* genes and three *SlRbcSs* genes, four soybean *GmRbcSs* genes and two *AtRbcSs* genes, five soybean *GmRbcSs* genes and three *NtRbcSs* genes, and five soybean *GmRbcSs* genes and three *MtRbcSs* genes, indicating that a limited number of *RbcSs* genes were conserved during evolution. Furthermore, the Ka/Ks ratio, a vital metric for inferring the evolutionary patterns of species selection, was computed. The results demonstrated that the ratio ranged from 0.14 to 0.28 between *GmRbcSs* and *AtRbcSs*, 0.19 to 0.33 between *GmRbcSs* and *MtRbcSs*, 0.13 to 0.22 between *GmRbcSs* and *NtRbcSs*, and 0.13 to 1.11 between *GmRbcSs* and *SlRbcSs* (Supplementary Table S3). These findings suggested that while a positive selection was observed between *GmRbcS7* and *SLRbcS3* (Ka/Ks > 1), the majority of genes primarily underwent purifying selection during evolution (Ka/Ks < 1), thus maintained relatively conserved functions.

### 2.5. Cis-Acting Elements Analysis of GmRbcSs Promoter

**Figure 5:**
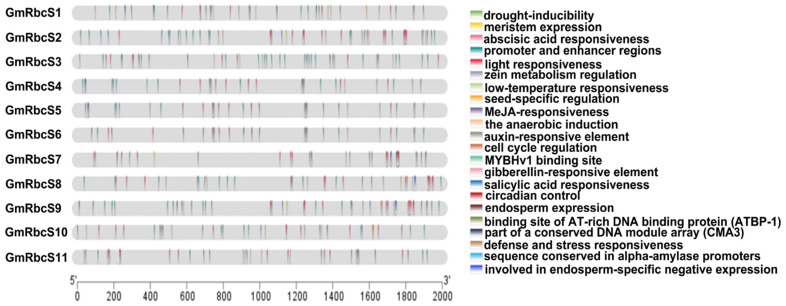
Cis-acting elements of the promoter region of 11 *GmRbcSs* genes.

To learn more about the function of the *GmRbcSs* family, possible cis-acting elements analysis was carried out on the promoter of these genes (Figure 5, Supplementary Table S4). Typically, the number of light-responsive elements was the largest and most widely distributed, totaling 145 in number. Notably, five hormone response elements were found, including those responsive to ABA, MeJA, SA, GA, and auxin, with a total count of 44 in 11 genes. Among them, multiple genes contained not just one hormone-responsive element, such as *GmRbcS1*, *GmRbcS2*, *GmRbcS4*, *GmRbcS5*, *GmRbcS6*, *GmRbcS7*, and *GmRbcS11*, suggesting that *GmRbcSs* might be implicated in multiple hormone response processes. There were also some biotic and abiotic stress response elements, such as the ones responsive to defense, low temperature, and drought, though their numbers were comparatively fewer. Additionally, many elements were involved in the regulatory processes among different tissues, such as meristem, endosperm, and seed, as well as in cell cycle regulation and differentiation during plant development. The number of cis-acting elements present within the promoters of diverse genes differed, which might be intimately linked to the functional specialization of these genes. For instance, *GmRbcS2* possessed the maximum cis-acting elements within the number of 183, whereas *GmRbcS6* contained the lowest count of 128. These findings indicated that the gene family might be involved in diverse physiological pathways in plants and possess a range of regulatory mechanisms under stress conditions.

### 2.6. Protein–Protein Interaction (PPI) Network in Soybean

**Figure 6:**
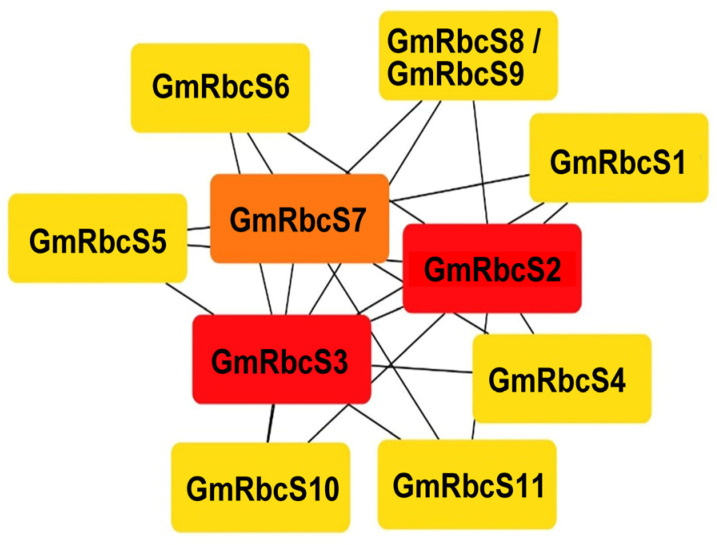
Interaction network among GmRbcSs protein.

The STRING database was utilized to predict potential interactions among GmRbcSs proteins. As shown in Figure 6, the GmRbcS protein interaction network comprised 10 nodes, with each node engaging in various interactions with the others. Notably, the interactions among these members were predominantly intricate multi-gene interactions. Among them, *GmRbcS2* and *GmRbcS3* were predicted to occupy a central position in the network, capable of radiating their influence on other genes.

### 2.7. Quantitative Analysis of GmRbcSs Genes in Different Tissues

**Figure 7:**
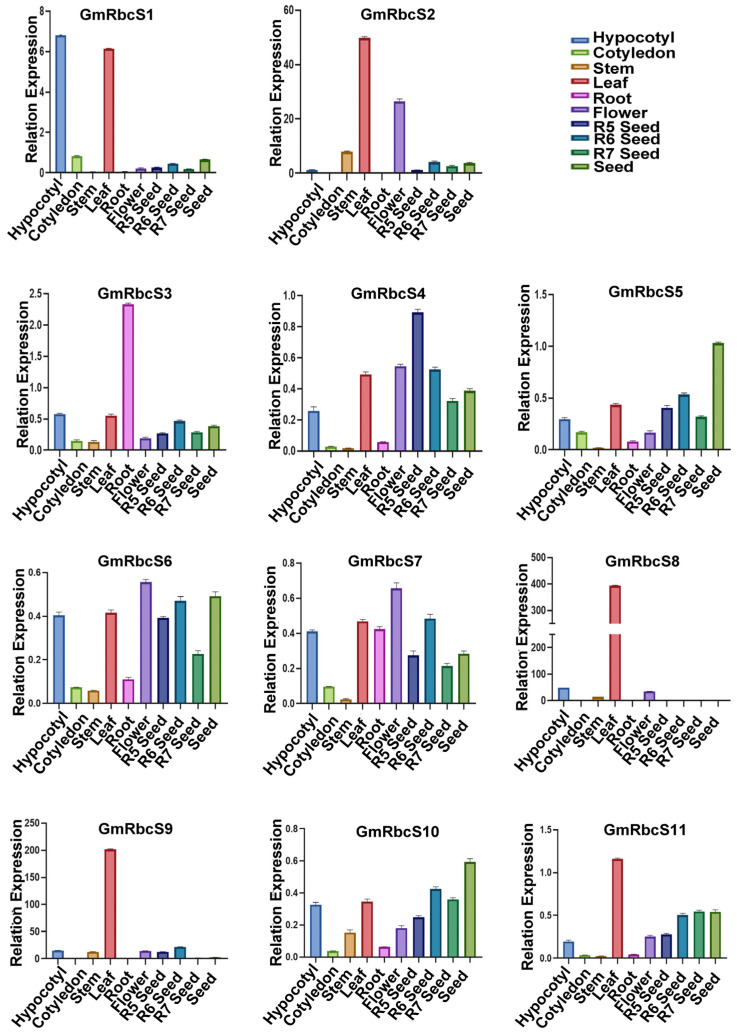
Expression pattern analysis of 11 *GmRbcSs* genes in diverse tissues and dynamic development seeds by RT-qPCR. Three biological replicates were analyzed per tissue.

To explore the expression pattern of the *GmRbcSs* gene family in different tissues of soybean, we analyzed the expression pattern of 11 genes among 10 tissues, including roots, hypocotyls, cotyledons, stems, leaves, flowers, and dynamic developing seeds by RT-qPCR. As depicted in Figure 7, the 11 genes exhibited significant differences in their expression patterns across the different tissues, with most *GmRbcSs* preferentially expressed in more than one tissue. Specifically, four genes showed the highest expression levels in leaves, two genes in flowers, two genes in seed, one gene in hypocotyl, one gene in the root, and one gene in the development seed of the R5 stage. Notably, *GmRbcS8* (*Glyma.19G046600*) exhibited the significantly highest relative expression level among the total 11 genes. These findings indicated that the *GmRbcSs* genes might play diverse roles in the growth and development process of soybeans.

### 2.8. Expression Patterns of GmRbcSs under Hormone Stresses

**Figure 8:**
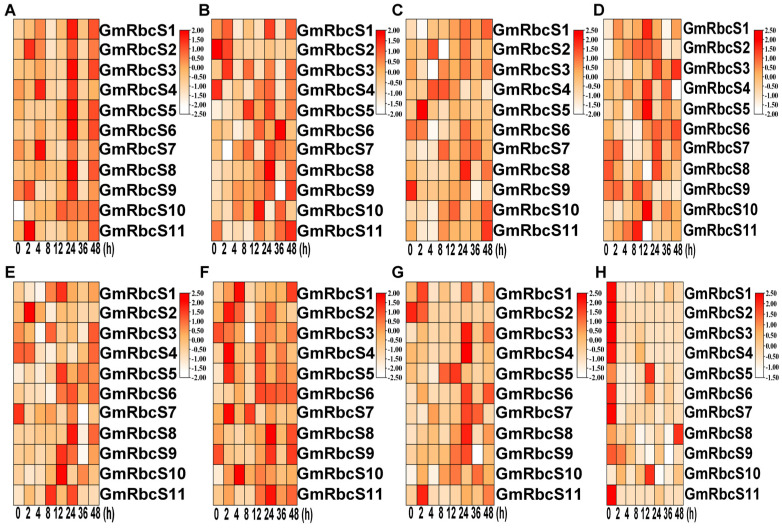
Expression heatmaps of the *GmRbcS* genes under different plant hormone treatments. The relative expression levels of genes are shown by the color gradient on the right scale for each hormone. The more intense the red color is, the higher the gene expression level, while the more intense the white color is, the lower the gene expression level. (**A**) BR, (**B**) MeJA, (**C**) ET, (**D**) IAA, (**E**) ABA, (**F**) SA, (**G**) trans-ZR, (**H**) GA3. Each bar value was the average value ± standard deviation based on three biological replicates.

Based on the presence of the cis-acting elements response to ABA, MeJA, SA, GA_3_, and auxin, the relative expression levels of *GmRbcSs* in leaves treated with not only the aforementioned hormones but also trans-ZR, ET, and BR were investigated using RT-qPCR to explore hormone-induced expression patterns. The results revealed that differential expressions of *GmRbcS* genes were exhibited under various hormone treatments within a period of 48 h (Figure 8). Compared to the control of 0 h, the expression patterns of the 11 genes displayed fluctuating changes in response to BR, with the exception of *GmRbcS10* (Figure 8A). When treated with MeJA, *GmRbcS2* and *GmRbcS4* exhibited a trend of down-regulation, whereas *GmRbcS5* and *GmRbcS8* showed up-regulation. Notably, the expression of *GmRbcS6* initially decreased and then increased with a peak point of 36 h. The remaining genes exhibited a fluctuating expression pattern throughout the observed period (Figure 8B). Similarly, the complexities of expression patterns among *GmRbcS* members were also observed in their responses to ET, IAA, ABA, SA, and trans-ZR. Among them, some genes were up-regulated, while others were down-regulated, and yet some genes exhibited fluctuations during the response period (Figure 8C–G). For instance, *GmRbcS8* was initially repressed and then up-regulated, reaching its highest expression level at 24 h in response to SA (Figure 8F). However, it showed a continuously significant up-regulated expression during the treatment period with trans-ZR, also peaking at 24 h (Figure 8G). Additionally, it was worth noting that the majority of genes consistently exhibited a specific decreasing expression level induced by GA_3_, with the exception of *GmRbcS5*, *GmRbcS8*, and *GmRbcS10*, whose expression level was decreased first and then increased (Figure 8H). Taken together, these findings demonstrated that a relatively intricate expression pattern existed among the *GmRbcS* genes in response to diverse plant hormones.

### 2.9. Transient Silencing GmRbcS8 Increases SMV Accumulation in Soybean

**Figure 9:**
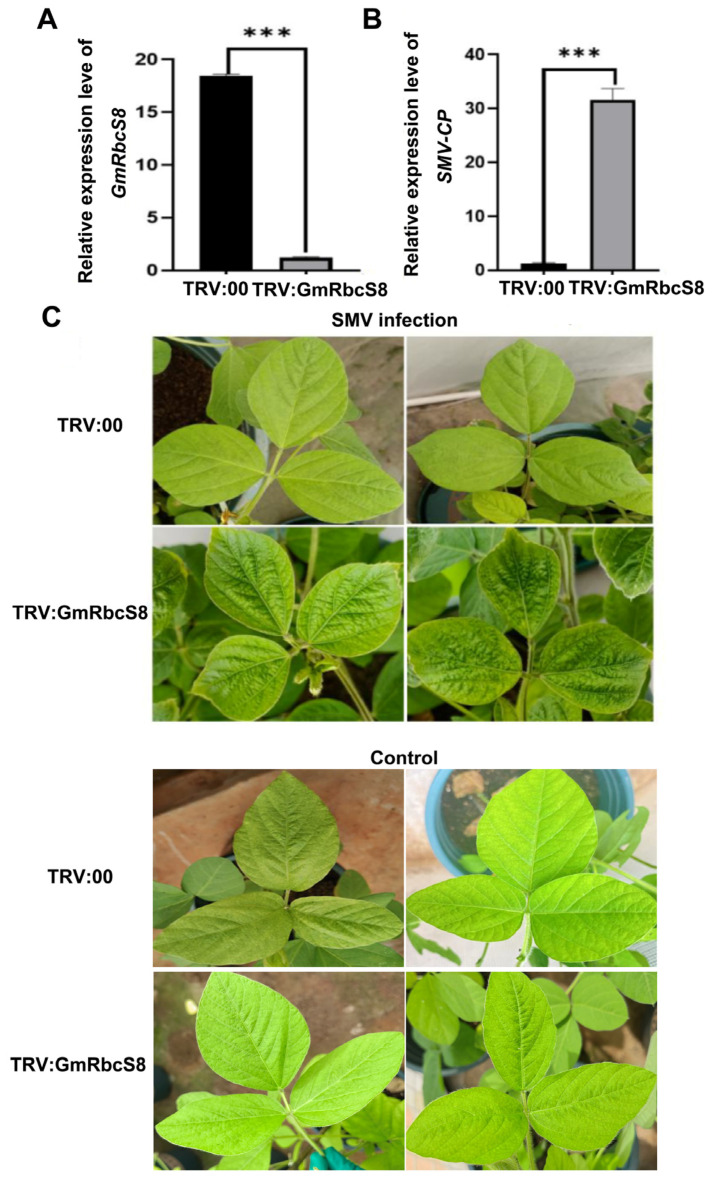
Functional analysis of *GmRbcS8* against SMV disease by VIGS. (**A**) Expression of *GmRbcS8* in silenced soybean leaves. (**B**) Expression level of *SMV-CP* gene in soybean leaves. (**C**) SMV phenotype in soybean leaves. *** indicates significant differences (*p* < 0.001).

Considering Rubisco played crucial roles in virus disease, we wondered whether *GmRbcSs* genes are involved in SMV response. Using the representative member *GmRbcS8* as an example, we silenced *GmRbcS8* in soybeans through the VIGS system and observed the subsequent SMV symptoms on leaves. The results indicated that the expression levels of *GmRbcS8* in the silenced soybeans (TRV:GmRbcS8) were significantly lower compared to the mock control (TRV:00), suggesting that *GmRbcS8* was effectively knocked down (Figure 9A). On this basis, the leaves of plants carrying TRV:GmRbcS8 and TRV:00 were inoculated with SMV, respectively, and the control group without SMV treatment was set up simultaneously. Upon SMV infection, the typical symptom, including leaf wrinkling, was clearly seen on the leaves of the silenced soybeans but not on those of SMV-inoculated control plants carrying TRV:00. The relative expression of the SMV coat protein (*CP*) gene was notably elevated in the silenced leaves (Figure 9B,C). Meanwhile, there were no differences in the leaf growth status of plants that were not treated with SMV (Figure 9C). These results demonstrated the ability of *GmRbcS8* to confer resistance of soybean to SMV, as well as the potential functions of *GmRbcSs* in response to SMV.

## 3. Discussion

Rubisco is the enzyme vital for carbon fixation in plants and is the most prevalent protein on Earth, consisting of eight large subunits (RbcL) and eight small subunits (RbcS) [42,43]. As crucial components, the RbcS subunits are positioned above and below the four RbcL dimers and are believed to play a role in assembling the large subunits and maintaining their structural integrity [20,44]. Notably, RbcS subunits are indirectly involved in the catalytic reaction, indicating their essential roles in plant growth and development, as well as responses to various abiotic stress [31,45,46,47]. Some RbcS proteins have been identified and functionally analyzed in Arabidopsis, tomato, wheat, rice, and cassava, but *RbcS* family genes have not yet been thoroughly characterized in soybeans [30,48,49,50,51]. In the present study, 11 *GmRbcS* genes were identified in soybeans, and their family members were comprehensively analyzed to explore the functions of the *GmRbcS* genes.

Genome comparisons among diverse organisms can provide rapid insights into their genomic structure and biological significance [52,53]. Previous studies have stated that the number of *RbcS* genes in different species varied, such as four members in Arabidopsis, five members in rice, and five members in tomato. In soybeans, the *RbcS* family comprises 11 members, indicating a larger number. The diversity in the amount of *RbcS* genes among species is primarily attributed to genome evolution and duplication, which causes the generation of homologous genes and novel genes, ultimately leading to their subsequent increase in number [51,54]. In the phylogenetic tree, 40 *RbcSs* from seven different species were classified into five clades, and three clades contained *GmRbcS* members (Figure 2). This classification aligned with previous research in rice and tomato, where *RbcS* genes were grouped into three distinct subfamilies [30,48]. According to the detailed molecular characterization of the GmRbcSs, members of the RbcS protein family had a variety of physicochemical characteristics, including amino acid length, MW, pI, instability index, and GRAVY values. This suggested a possible degree of diversity in physiological properties among these family members. Furthermore, the 11 *GmRbcS* genes shared conserved domain characteristics of the Rubisco small subunit and similar motifs and comparable exon–intron counts. Taken together, these findings implied that members of the *GmRbcS* family might exhibit both similarity and differentiation in various functional processes.

Cis-acting elements of promoters play crucial roles in regulating gene expression during biological processes such as growth and development, stress response, and signal transduction [55]. In this study, several elements were identified to be associated with biotic stress (defense and stress element), abiotic stress (low temperature and drought), and plant development (meristem expression, seed-specific regulation, cell cycle regulation, etc.). Additionally, a significant proportion of the elements was also found to be responsive to growth hormones (GA, auxin, and ABA) and defense hormones (MeJA and SA) regulatory pathways, although light responsiveness was observed to be the most numerous. This suggested that the expressions of *GmRbcS* genes might be intimately linked to plant development and stress responses (Figure 5, Supplementary Table S4). In transgenic tobacco, spatial and light regulation of the soybean *RbcS* promoter has been identified, indicating its specificity to leaves and inducibility by light [56]. Silencing of *NbRbcS* enabled Tomato mosaic tobamovirus (ToMV) to cause necrosis in inoculated leaves, effectively enhancing the local infectivity of the virus [57]. *OsRbcS3* could improve rice chilling tolerance at the seedling and booting stage [47]. Taken together, the existence of specific cis-acting elements underscores the significance of *GmRbcS* for regulating gene expression and adapting to diverse environmental conditions.

Tissue-specific expression patterns have been considered as one of the effective approaches to predict genes involved in specific processes of plant development to a certain extent. In this study, the expression of 11 *GmRbcS* members was analyzed in multiple tissues and dynamic stages of developing seeds (Figure 7). Among them, most genes displayed specific high expression in trifoliate leaves, including *GmRbcS2*, *GmRbcS8*, *GmRbcS9*, and *GmRbcS11*, whose patterns were similar to the expression of four genes (*OsRBCS2*, *OsRBCS3*, *OsRBCS4*, and *OsRBCS5*) in rice [30] and two genes (*RBCS1A* and *RBCS3B*) in Arabidopsis [31], suggesting a possibly predominant role in soybean leaves. Two genes (*GmRbcS6* and *GmRbcS7*) exhibited the highest expression level in flowers, while *GmRbcS5* and *GmRbcS10* showed the highest expression in seed, and *GmRbcS4* displayed the highest expression specifically in the stage of R5 seed, which indicated that these genes might play a role in the reproductive growth of soybean. Additionally, *GmRbcS1* had the highest expression level in the hypocotyl, and *GmRbcS3* showed a specific high expression in the root. These results demonstrated that *GmRbcS* genes possessed spatiotemporal expression characteristics in a tissue-specific manner, especially in photosynthetic organs, which was due to the fact that transit peptides target proteins to chloroplasts and mitochondria [58,59,60]. These findings indicated that the *GmRbcS* genes might play a variety of roles in the development process of soybeans.

Plant hormones, a group of small signaling molecules, are the major players in regulating plant growth, development, and response to environmental stresses [61,62]. In this study, the *GmRbcSs* genes showed varying degrees of responsiveness to plant hormones during the 48 h treatment period (Figure 8). For instance, most genes demonstrated a decreased expression level in response to GA_3_. Considering the potential abilities of GA_3_ to induce seed germination and some abiotic stresses [63,64], the results indicated that *GmRbcSs* might play a role in regulating the soybean seed characteristics. Furthermore, numerous genes were significantly detected as sensitive to BR, displaying either increased or decreased expression levels during the treatment period. However, *GmRbcS10* was persistently up-regulated with the peak of 12 h and exhibited apparent differences in expression levels in response to BR. The defense hormones, including MeJA and SA, have been shown to induce the expression of numerous genes, exhibiting a notable and dynamic pattern of expression. MeJA and SA usually acted as elicitors in mediating plant responses to biotic stresses [65,66], suggesting the crucial role of *GmRbcS* genes in plant disease. A similar expression pattern was observed among many genes after trans-ZR treatment, the most active and ubiquitous cytokinin, which could crosstalk with other hormones involved in promoting plant development and enhancing plant immunity [67,68]. Notably, *GmRbcS8* exhibited a relatively durable and significantly high expression to trans-ZR and MeJA compared with other members and harbored the same expression trend with the peak at 24 h, indicating that *GmRbcS8* might be a key factor in balancing plant growth and defense based on the contents changing between trans-ZR and MeJA. Taken together, all these expression patterns highlighted the complexity and diversity of *GmRbcS* genes in their responses to various plant hormones, thereby playing regulatory functions in plant development and defense mechanisms.

To date, many studies have verified that RbcS is closely associated with the process of plant virus response, especially in the *Potyviridae* family. The function of onion RbcS was disturbed by its interaction with both P3 and P3N-PIPO in cases of several potyviruses; rubisco was then subsequently influenced and finally induced the viruses’ developments [40]. In recent research, the reduction of *NbRbCS* levels greatly impaired the intercellular movement of four potyvirids, indicating RbCS was selected by the virus as a scaffold protein to enable viral movement [42]. These findings suggest that RbcS could not only physically interact with virus proteins but also act as a scaffold protein to participate in the process of viral movement in host plants. Additionally, using a transient expression assay, Zhao et al. (2013) identified that *NbRbCS* played a vital role in tobamovirus movement and plant antiviral defenses by silencing *NbRbCS* and observing local and systemic viral symptoms on leaves [57]. Here, to identify whether *GmRbcS* members could participate in the response to SMV, the most destructive viral disease affecting soybeans [43], we employed the VIGS assay to silence *GmRbcS8* (*Glyma.19G046600*), whose expression abundance was the highest among all *GmRbcS* genes in soybean leaves. Notably, more serious SMV-susceptible symptoms were observed on soybean leaves with the silenced *GmRbcS8* gene compared to the control group, suggesting its key role in responding to SMV disease.

## 4. Materials and Methods

### 4.1. Characterization and Physicochemical Properties of the RbcS Family in Soybean

All candidate RbcS proteins in the soybean genome were searched using the Phytozome database (https://phytozome-next.jgi.doe.gov/, accessed on 16 May 2024) with the RbcS proteins of *Arabidopsis thaliana* (*A. thaliana*) as a reference. The Hidden Markov Model (HMM) file for RbcS proteins was downloaded from the PFAM protein family database (http://pfam.xfam.org/, accessed on 16 May 2024) within the Pfam number PF12338. This HMM file was then utilized as input for HMMER-3.1b2 software (http://hmmer.janelia.org/, accessed on 16 May 2024) to retain the protein sequences [69]. The NCBI-CDD (https://www.ncbi.nlm.nih.gov/Structure/, accessed on 16 May 2024) and SMART (http://smart.embl-heidelberg.de/, accessed on 16 May 2024) databases were used to validate these proteins [70,71]. Furthermore, the physical and chemical properties of the soybean RbcS using the online program ExPasy (http://web.expasy.org/protparam/, accessed on 20 May 2024) were identified. These properties included sequence length, protein molecular weight, and other pertinent parameters. Additionally, the subcellular localization of the GmRbcS proteins was predicted using the WoLF PSORT website (https://wolfpsort.hgc.jp/, accessed on 26 May 2024).

### 4.2. Phylogenetic and Structure Analysis

The RbcS protein sequences of *A. thaliana*, *Nicotiana benthamian* (*N. benthamiana*), *Medicago truncatula* (*M. truncatula*), tomato, rice, and sorghum, were found from the phytozome database (https://phytozome-next.jgi.doe.gov/, accessed on 16 May 2024). The HMM file and NCBI-CDD were used to validate these proteins. Then, the ClustalW algorithm was used to compare and analyze the RbcS protein sequences of these seven species. The phylogenetic tree was constructed based on the neighborhood method in MEGA7.0 software, and the bootstrap value was set to 1000. The final visualization was performed using the iTOL website (https://itol.embl.de/login.cgi?logout=1, accessed on 27 May 2024). Subsequently, the structure of GmRbcS was analyzed, and the location and quantity of exons and introns were obtained by GSDS2.0 (http://gsds.gao-lab.org/index.php, accessed on 24 May 2024). Simultaneously, the conserved motif of the GmRbcS protein was predicted, and visual analysis was performed via the MEME website (https://meme-suite.org/meme/tools/meme, accessed on 24 May 2024). Finally, TBtools software (Tbtools, version 1.098) was used to analyze the development of the visual system.

### 4.3. Chromosomal Mapping and Gene Replication

Utilizing the soybean genome annotation file, the chromosome location information of the *RbcS* genes was extracted and visualized using MapChart software (A plugin in Tbtools, version 1.098). The Multiple Collinearity Scan toolkit (MCScanX) was used to investigate the repetitive events of *RbcS* genes in soybeans, and the results were visualized via TBtools. Additionally, the KaKs_Calculator 2.0 was utilized to determine the nonsynonymous (Ka) and synonymous (Ks) substitutions, as well as the Ka/Ks ratio, specifically for the segmental duplication gene pairs [72].

### 4.4. Gene Regulatory Network Analysis

STRING online software (https://STRING-db.org/, accessed on 28 May 2024) was used to predict the protein–protein interactions among GmRbcS proteins. Cytoscape 3.7.2 was then used to optimize the protein–protein interaction network [73].

### 4.5. Analysis of Cis-Acting Elements in Promoter Sequences

The 2.0 kb upstream sequences of *GmRbcS* family genes were downloaded from the Phytozome database (https://phytozome-next.jgi.doe.gov/, accessed on 28 May 2024) and then submitted to the PlantCARE website (http://bioinformatics.psb.ugent.be/webtools/plantcare/html/, accessed on 28 May 2024). The diverse cis-regulatory elements in each gene were identified and visualized by TBtools software.

### 4.6. Spatiotemporal Expression Analysis

Tissue-specific spatiotemporal expression analysis was conducted on soybean cultivar ‘Williams 82’ using real-time quantitative PCR (RT-qPCR). Roots, hypocotyls, cotyledons, stems, trifoliate soybean leaves, flowers, and dynamic development seeds at the reproductive growth stage of R5, R6, R7, and R8 were collected, respectively. For each tissue type, three biological replicates were set up.

### 4.7. Soybean Materials and Hormone Treatments

The four-week-old seedlings of Williams 82 soybeans were treated with Hoagland solution medium containing 100 μM gibberellin (GA), 100 μM auxin, 50 μM abscisic acid (ABA), 50 μM brassinosteroid (BR), 50 μM Methyl jasmonate (MeJA), 50 μM salicylic acid (SA), 50 μM ethylene (ET), and 50 μM trans-Zeatin (tZ), respectively. The leaves were sprayed at 0 h, 2 h, 4 h, 8 h, 12 h, 24 h, 36 h, and 48 h, respectively, and sampling was completed uniformly. At each time point, three different plants were sampled to set three biological replicates and three leaves were taken from each plant as samples.

### 4.8. Total RNA Extraction and RT-qPCR Analysis

Total RNA was extracted using Triozol (Invitrogen, Carlsbad, CA, USA), and then the first chain of cDNA was obtained by M5 Sprint plus qPCR RT kit with gDNA remover (Mei5bio, Beijing, China). Specific primers for the eleven *GmRbcS* genes were designed with Primer 5 software; 2X M5 HiPer SYBR Premix EsTaq (with Tli RnaseH) (Mei5bio, China) was used for standard expression detection, and the experiment was repeated three times with *GmACTIN4* as a normalization control. The reaction procedure was 95 °C, 3 min, followed by 40 cycles of 95 °C for 5 s and 60 °C for 30 s; the dissolution curve was 95 °C, 20 s; 60 °C, 30 s; 95 °C, 20 s. RT-qPCR was conducted with three biological replicates and three technical repeats for each sample. The relative expression levels were calculated by the 2^−ΔΔCt^ method. To evaluate the accuracy of the verification, the Student’s *t*-test was used to assess whether these differences were statistically significant, including * *p* < 0.05, ** *p* < 0.01, and *** *p* < 0.001. Primer pairs used in this study are listed in Supplementary Table S5.

### 4.9. Virus-Induced Gene Silencing System (VIGS)

The VIGS technique was performed for the knock-down of the *GmRbcS8* gene, according to the previous approaches [74,75]. To create the TRV2:GmRbcS8 vector, a 279 bp CDS fragment was amplified using the specific primer pair with the restriction enzyme site of *Xba* I. *GmRbcS8* was cloned into the TRV2 vector, while the TRV2:00 vector was used as a negative control. Then, recombinant plasmids were introduced into *Agrobacterium tumefaciens* strain GV3101 (pSoup-p19). Agrobacterium cells were harvested and activated in an infiltration buffer adjusted to an optical density at 600 nm of 1.0, and the suspensions were infiltrated into the fully extended soybean leaves using a needleless 1 mL syringe. Approximately 10 days later, leaf samples from the silenced plants and control plants were collected. The silencing efficiency was then estimated by determining the relative expression level of *GmRbcS8* through the RT-qPCR assay with three biological repetitions.

### 4.10. Assessing the Response of Silenced GmRbcS8 Gene to SMV

Artificial SMV inoculation was performed using a previously described method with minor adjustments [76]. In short, soybean leaves infected with the SMV-G3 strain were ground with emery powder, and an appropriate amount of 0.01 mol·L^−1^ phosphate buffer was added to prepare the virus solution. Using a brush dipped in the virus abrasive solution, the second trifoliate soybean leaves of *GmRbcS8*-silenced plants and the control group were evenly coated using friction inoculation, respectively. Two weeks later, total RNA was extracted from the uninoculated upper leaves, and the expression level of the SMV coat protein gene *CP* in silenced plants was detected by RT-qPCR assay. The biological replicates were repeated three times.

## 5. Conclusions

In this study, a total of 11 *RbcS* members of soybean were identified. A comprehensive analysis was first conducted aiming to understand the characteristics of these *GmRbcS* genes, encompassing physicochemical characteristics, gene structure analysis, chromosomal distributions, evolutionary relationships, cis-acting elements, etc. Secondly, the spatiotemporal expression patterns of *GmRbcSs* among tissues and response to plant hormones were further characterized by RT-qPCR, revealing their extensive involvement in responses to various plant hormones. Finally, the identification of the SMV-responsive gene *GmRbcS8* was performed to evaluate the resistance function of *GmRbcS8* through the VIGS assay, suggesting that *GmRbcSs* might play an important role in regulating SMV infection in the host. In conclusion, this study provides a theoretical basis and lays the foundation for the function and regulatory mechanisms of *RbcS* genes in soybeans. Further research will be devoted to fully elucidating their functions and roles in plant growth, development, and stress responses.

## Figures and Tables

**Table 1:** Characteristics of *GmRbcSs* family members.

Gene ID	Gene Name	Protein Length (aa)	Molecular Weight (kDa)	Theoretical Isoelectric Point (pI)	Instability Index	GRAVY	Subcellular Location
Glyma.08G365300	GmRbcS1	90	9.97	8.89	43.52	−0.12	Chloroplast
Glyma.13G046200	GmRbcS2	178	20.02	8.87	30.71	−0.24	Chloroplast
Glyma.13G097100	GmRbcS3	172	19.60	8.95	53.39	−0.35	Chloroplast
Glyma.14G089500	GmRbcS4	159	17.83	9.81	45.98	−0.53	Chloroplast
Glyma.18G182200	GmRbcS5	100	11.54	9.17	49.22	−0.33	Chloroplast
Glyma.18G182300	GmRbcS6	131	15.09	9.20	50.32	−0.48	Chloroplast
Glyma.18G296900	GmRbcS7	150	16.76	9.22	43.77	−0.14	Chloroplast
Glyma.19G046600	GmRbcS8	178	20.01	8.87	31.32	−0.24	Chloroplast
Glyma.19G046800	GmRbcS9	178	20.01	8.87	31.32	−0.24	Chloroplast
Glyma.19G046900	GmRbcS10	135	14.81	9.39	33.23	−0.06	Chloroplast
Glyma.19G047000	GmRbcS11	105	12.41	7.81	36.75	−0.45	Chloroplast

## Data Availability

Data are contained within the article and Supplementary Materials.

## Supplementary Materials

The following supporting information can be downloaded at: https://www.mdpi.com/article/10.3390/ijms25179231/s1.
